# Supplementary material for: Inbreeding in a dioecious plant has sex- and population origin-specific effects on its interactions with pollinators
Source: eLife. 2021 May 14;10:e65610. doi: 10.7554/eLife.65610 (PMC8159375; doi:10.7554/eLife.65610)
Supplement: Supplementary file 1. — The 20 VOC, which evidently trigger antennal responses in Hadena bicruris (Dötterl et al., 2006) and were thus analysed as a sub-dataset, are highlighted in bold. [file elife-65610-supp1.docx]

**Supplementary File 1:** Mean ± SE abundance (e-06) of 70 volatile organic compounds (VOC) emitted by *Silene latifolia* plants in all breeding treatment × sex × origin combinations, as determined by silicone tubing headspace collection combined with thermo desorption–gas chromatography–mass spectrometry. The 20 VOC, which evidently trigger antennal responses in *Hadena bicruris* (Dötterl et al., 2006) and were thus analysed as a sub-dataset, are highlighted in bold.

| Compound class | Putative identity | Europe | | | | | | | | | | | | North America | | | | | | | | | | | |
| --- | --- | --- | --- | --- | --- | --- | --- | --- | --- | --- | --- | --- | --- | --- | --- | --- | --- | --- | --- | --- | --- | --- | --- | --- | --- |
|  |  | Female | | | | | | Male | | | | | | Female | | | | | | Male | | | | | |
|  |  | Outbred | | | Inbred | | | Outbred | | | Inbred | | | Outbred | | | Inbred | | | Outbred | | | Inbred | | |
| Aldehyde | **Nonanal** | 11.6 | ± | 1.2 | 12.1 | ± | 1.0 | 14.1 | ± | 0.9 | 13.4 | ± | 1.1 | 12.8 | ± | 0.6 | 12.5 | ± | 0.5 | 11.1 | ± | 1.0 | 13.0 | ± | 0.9 |
|  | (*E*)-2-Nonenal | 0.0 | ± | 0.0 | 0.0 | ± | 0.0 | 0.0 | ± | 0.0 | 0.0 | ± | 0.0 | 0.1 | ± | 0.1 | 0.0 | ± | 0.0 | 0.2 | ± | 0.1 | 0.0 | ± | 0.0 |
|  | **Decanal** | 6.3 | ± | 0.6 | 5.7 | ± | 0.7 | 5.3 | ± | 0.7 | 5.6 | ± | 0.8 | 6.2 | ± | 0.5 | 6.7 | ± | 0.4 | 6.2 | ± | 0.5 | 6.6 | ± | 0.5 |
|  | 5-Hydroxymethylfurfural | 2.0 | ± | 1.4 | 0.7 | ± | 0.5 | 4.9 | ± | 2.2 | 12.1 | ± | 6.3 | 0.2 | ± | 0.1 | 5.8 | ± | 4.8 | 2.9 | ± | 1.1 | 4.4 | ± | 2.0 |
|  | Undecanal | 0.4 | ± | 0.2 | 0.2 | ± | 0.2 | 0.6 | ± | 0.3 | 0.3 | ± | 0.2 | 0.0 | ± | 0.0 | 0.1 | ± | 0.1 | 0.3 | ± | 0.2 | 0.1 | ± | 0.1 |
|  | Dodecanal | 2.7 | ± | 0.2 | 2.9 | ± | 0.1 | 3.0 | ± | 0.1 | 2.7 | ± | 0.1 | 2.8 | ± | 0.1 | 2.8 | ± | 0.1 | 2.7 | ± | 0.1 | 2.9 | ± | 0.1 |
|  | Tetradecanal | 3.6 | ± | 0.2 | 3.4 | ± | 0.2 | 3.4 | ± | 0.2 | 3.2 | ± | 0.2 | 3.1 | ± | 0.2 | 3.5 | ± | 0.2 | 3.3 | ± | 0.2 | 3.5 | ± | 0.2 |
| Alkane | 2,2,4-Trimethylheptane | 117.1 | ± | 24.2 | 110.6 | ± | 22.2 | 94.2 | ± | 21.4 | 89.4 | ± | 18.5 | 124.4 | ± | 24.5 | 118.5 | ± | 26.3 | 116.2 | ± | 24.7 | 142.7 | ± | 22.4 |
|  | Undecane | 27.8 | ± | 5.0 | 32.6 | ± | 4.9 | 32.5 | ± | 6.2 | 28.6 | ± | 4.5 | 18.5 | ± | 1.8 | 19.7 | ± | 2.9 | 20.2 | ± | 3.6 | 23.8 | ± | 4.6 |
|  | Hexadecane | 6.2 | ± | 1.0 | 7.0 | ± | 0.7 | 6.3 | ± | 1.0 | 7.2 | ± | 1.1 | 4.6 | ± | 0.7 | 5.4 | ± | 0.7 | 5.7 | ± | 0.6 | 5.6 | ± | 0.6 |
|  | 2,6,8-Trimethyltridecane | 7.6 | ± | 0.7 | 7.4 | ± | 0.6 | 8.0 | ± | 0.9 | 8.0 | ± | 1.0 | 6.4 | ± | 0.5 | 6.4 | ± | 0.6 | 6.4 | ± | 0.5 | 6.4 | ± | 0.5 |
|  | Tetracedane | 3.9 | ± | 0.2 | 4.3 | ± | 0.5 | 4.3 | ± | 0.3 | 4.1 | ± | 0.3 | 3.9 | ± | 0.1 | 3.4 | ± | 0.3 | 3.6 | ± | 0.2 | 3.8 | ± | 0.1 |
|  | Pentadecane | 3.9 | ± | 0.3 | 4.5 | ± | 0.5 | 4.8 | ± | 0.7 | 4.1 | ± | 0.3 | 3.7 | ± | 0.2 | 4.0 | ± | 0.3 | 3.8 | ± | 0.1 | 4.0 | ± | 0.3 |
|  | Octadecane | 2.6 | ± | 1.2 | 3.5 | ± | 1.2 | 1.0 | ± | 0.7 | 2.1 | ± | 1.4 | 3.3 | ± | 1.3 | 2.9 | ± | 1.2 | 4.0 | ± | 1.3 | 4.9 | ± | 1.6 |
| Alkohol | (*Z*)-3-Nonen-1-ol | 0.6 | ± | 0.4 | 0.8 | ± | 0.3 | 0.7 | ± | 0.4 | 0.9 | ± | 0.4 | 2.5 | ± | 0.7 | 1.3 | ± | 0.4 | 2.5 | ± | 0.6 | 1.1 | ± | 0.4 |
|  | 2-Methyl-1-decanol | 3.6 | ± | 0.2 | 3.9 | ± | 0.2 | 4.1 | ± | 0.5 | 4.1 | ± | 0.3 | 3.5 | ± | 0.2 | 3.4 | ± | 0.2 | 3.5 | ± | 0.2 | 3.6 | ± | 0.2 |
| Benzenoid | **Benzaldehyde** | 25.0 | ± | 8.1 | 14.7 | ± | 3.6 | 9.9 | ± | 2.1 | 9.8 | ± | 1.9 | 14.2 | ± | 2.3 | 17.8 | ± | 2.8 | 14.3 | ± | 3.4 | 12.8 | ± | 2.4 |
|  | Benzyl alcohol | 21.2 | ± | 9.1 | 12.6 | ± | 3.9 | 4.9 | ± | 1.1 | 20.5 | ± | 5.6 | 20.2 | ± | 5.3 | 14.3 | ± | 4.2 | 13.9 | ± | 5.3 | 5.1 | ± | 0.8 |
|  | **2-Phenylacetaldehyde** | 13.6 | ± | 6.3 | 9.4 | ± | 3.1 | 17.3 | ± | 5.5 | 12.9 | ± | 6.3 | 15.5 | ± | 5.5 | 32.4 | ± | 10.6 | 20.5 | ± | 6.4 | 21.2 | ± | 6.7 |
|  | **Methylbenzoate** | 2.2 | ± | 0.6 | 5.9 | ± | 3.4 | 3.1 | ± | 0.7 | 5.2 | ± | 2.1 | 3.9 | ± | 1.2 | 5.3 | ± | 2.4 | 8.1 | ± | 6.0 | 2.1 | ± | 0.4 |
|  | **Benzyl acetate** | 10.9 | ± | 4.8 | 9.1 | ± | 5.5 | 13.7 | ± | 7.4 | 12.1 | ± | 7.7 | 7.2 | ± | 5.4 | 3.5 | ± | 1.8 | 17.0 | ± | 7.7 | 12.9 | ± | 5.4 |
|  | **Methylsalicylate** | 10.2 | ± | 7.0 | 9.6 | ± | 6.6 | 12.9 | ± | 8.9 | 22.0 | ± | 10.4 | 21.1 | ± | 9.9 | 28.4 | ± | 11.6 | 24.0 | ± | 11.3 | 6.4 | ± | 6.4 |
| Carboxylic acid | Nonanoic acid | 5.3 | ± | 1.0 | 5.4 | ± | 0.9 | 4.5 | ± | 1.0 | 5.0 | ± | 1.1 | 5.6 | ± | 1.0 | 5.3 | ± | 0.8 | 4.8 | ± | 0.7 | 6.0 | ± | 0.7 |
| Ester | 2-Tetrahydro furanmethanol | 47.9 | ± | 18.5 | 40.9 | ± | 19.2 | 57.3 | ± | 21.0 | 46.3 | ± | 17.0 | 78.8 | ± | 23.3 | 94.2 | ± | 25.4 | 75.3 | ± | 21.8 | 78.9 | ± | 22.7 |
|  | Octen-3-ol | 0.8 | ± | 0.2 | 0.5 | ± | 0.1 | 0.8 | ± | 0.2 | 0.7 | ± | 0.2 | 0.4 | ± | 0.1 | 0.4 | ± | 0.1 | 0.6 | ± | 0.2 | 0.5 | ± | 0.1 |
|  | **Ethylhexanoate** | 1.5 | ± | 0.3 | 1.3 | ± | 0.4 | 1.5 | ± | 0.3 | 1.8 | ± | 0.4 | 0.9 | ± | 0.3 | 1.4 | ± | 0.4 | 1.3 | ± | 0.4 | 1.6 | ± | 0.4 |
|  | Hexyl pentanoate | 0.3 | ± | 0.2 | 0.0 | ± | 0.0 | 0.2 | ± | 0.2 | 0.5 | ± | 0.5 | 0.4 | ± | 0.3 | 0.6 | ± | 0.4 | 0.6 | ± | 0.4 | 0.1 | ± | 0.1 |
|  | (*Z*)-3-Hexenyl acetate | 0.7 | ± | 0.6 | 0.3 | ± | 0.2 | 2.5 | ± | 0.9 | 1.8 | ± | 1.1 | 0.2 | ± | 0.2 | 0.7 | ± | 0.6 | 0.8 | ± | 0.6 | 0.3 | ± | 0.2 |
| Fatty acid derivate | **(*E*)-3-Hexenol** | 0.6 | ± | 0.3 | 0.5 | ± | 0.3 | 0.5 | ± | 0.3 | 0.9 | ± | 0.4 | 0.3 | ± | 0.2 | 1.4 | ± | 0.7 | 0.4 | ± | 0.3 | 0.4 | ± | 0.2 |
|  | (*Z*)-2-Hexen-1-ol | 69.7 | ± | 17.0 | 85.5 | ± | 20.5 | 73.0 | ± | 17.6 | 77.1 | ± | 17.1 | 116.3 | ± | 21.8 | 125.1 | ± | 23.3 | 96.1 | ± | 19.8 | 114.5 | ± | 20.6 |
| Ketone | Pimelic ketone | 1.5 | ± | 0.4 | 1.5 | ± | 0.4 | 1.2 | ± | 0.4 | 2.0 | ± | 0.5 | 0.8 | ± | 0.4 | 1.3 | ± | 0.5 | 1.5 | ± | 0.4 | 1.6 | ± | 0.4 |
|  | 2-Heptanone | 37.2 | ± | 4.5 | 40.0 | ± | 4.5 | 36.3 | ± | 4.2 | 40.2 | ± | 4.5 | 39.9 | ± | 3.4 | 45.6 | ± | 3.6 | 39.4 | ± | 3.4 | 35.1 | ± | 2.9 |
|  | Butyrophenone | 1.0 | ± | 0.3 | 1.6 | ± | 0.8 | 0.3 | ± | 0.1 | 0.7 | ± | 0.4 | 1.3 | ± | 0.5 | 1.3 | ± | 0.6 | 0.9 | ± | 0.4 | 0.8 | ± | 0.5 |
| Mono-terpenoid | α-Pinene | 158.6 | ± | 22.4 | 164.9 | ± | 21.6 | 196.9 | ± | 17.9 | 160.2 | ± | 26.0 | 174.3 | ± | 19.6 | 163.4 | ± | 17.8 | 156.6 | ± | 20.8 | 122.3 | ± | 21.4 |
|  | Camphene | 26.0 | ± | 3.2 | 25.6 | ± | 3.7 | 25.9 | ± | 3.3 | 30.9 | ± | 4.0 | 22.6 | ± | 3.6 | 27.6 | ± | 3.7 | 25.7 | ± | 3.4 | 28.8 | ± | 4.1 |
|  | β-Pinene | 3.7 | ± | 0.4 | 3.7 | ± | 0.5 | 3.8 | ± | 0.5 | 4.3 | ± | 0.6 | 3.2 | ± | 0.5 | 3.9 | ± | 0.5 | 3.7 | ± | 0.5 | 4.0 | ± | 0.6 |
|  | **ß-Myrcene** | 14.9 | ± | 2.1 | 14.8 | ± | 2.7 | 15.2 | ± | 2.4 | 18.7 | ± | 2.8 | 12.6 | ± | 2.4 | 15.9 | ± | 2.7 | 14.7 | ± | 2.4 | 16.7 | ± | 2.8 |
|  | **p-Cymene** | 8.1 | ± | 1.1 | 8.1 | ± | 1.4 | 8.1 | ± | 1.2 | 10.1 | ± | 1.4 | 6.9 | ± | 1.2 | 8.7 | ± | 1.4 | 8.1 | ± | 1.3 | 9.2 | ± | 1.4 |
|  | d-Limonene | 30.2 | ± | 4.2 | 30.3 | ± | 5.4 | 31.3 | ± | 5.0 | 37.8 | ± | 5.6 | 24.3 | ± | 4.8 | 31.0 | ± | 5.4 | 28.5 | ± | 4.7 | 33.3 | ± | 5.6 |
|  | Eucalyptol | 17.6 | ± | 4.7 | 31.3 | ± | 7.9 | 18.0 | ± | 5.4 | 16.1 | ± | 4.4 | 19.9 | ± | 4.2 | 16.8 | ± | 3.6 | 14.5 | ± | 3.4 | 18.5 | ± | 3.9 |
|  | **(*Z*)-β-Ocimene** | 1.0 | ± | 0.7 | 1.9 | ± | 0.8 | 1.0 | ± | 0.6 | 0.1 | ± | 0.1 | 1.8 | ± | 0.9 | 1.0 | ± | 0.6 | 2.8 | ± | 1.3 | 0.2 | ± | 0.2 |
|  | g-Terpinene | 8.3 | ± | 1.2 | 8.2 | ± | 1.6 | 8.6 | ± | 1.4 | 10.4 | ± | 1.6 | 6.2 | ± | 1.3 | 8.2 | ± | 1.5 | 7.4 | ± | 1.3 | 8.8 | ± | 1.6 |
|  | **Lilac aldehyde A** | 22.2 | ± | 5.0 | 33.6 | ± | 7.6 | 35.2 | ± | 7.4 | 30.0 | ± | 6.7 | 42.5 | ± | 8.5 | 26.1 | ± | 6.3 | 30.0 | ± | 4.3 | 42.1 | ± | 6.7 |
|  | **Lilac aldehyde B/C** | 48.3 | ± | 11.9 | 68.9 | ± | 15.2 | 72.5 | ± | 15.7 | 78.8 | ± | 16.9 | 70.6 | ± | 14.3 | 54.8 | ± | 13.4 | 51.8 | ± | 8.0 | 67.0 | ± | 12.1 |
|  | **Lilac aldehyde D** | 4.2 | ± | 2.2 | 10.0 | ± | 3.6 | 2.8 | ± | 1.0 | 6.9 | ± | 1.6 | 9.2 | ± | 3.7 | 5.4 | ± | 2.4 | 3.6 | ± | 1.5 | 4.9 | ± | 1.5 |
|  | Methyl isoborneol | 0.0 | ± | 0.0 | 0.2 | ± | 0.1 | 0.2 | ± | 0.1 | 0.2 | ± | 0.1 | 0.1 | ± | 0.1 | 0.0 | ± | 0.0 | 0.0 | ± | 0.0 | 0.1 | ± | 0.1 |
|  | Lilac alcohol a | 2.6 | ± | 1.8 | 6.0 | ± | 4.4 | 1.6 | ± | 0.7 | 4.2 | ± | 2.0 | 1.5 | ± | 1.4 | 0.4 | ± | 0.3 | 2.5 | ± | 1.3 | 1.1 | ± | 0.5 |
|  | **Lilac alcohol B/C** | 3.3 | ± | 1.7 | 5.2 | ± | 3.1 | 5.2 | ± | 3.3 | 4.7 | ± | 1.8 | 3.2 | ± | 0.8 | 2.6 | ± | 1.0 | 5.9 | ± | 2.4 | 2.2 | ± | 0.5 |
|  | Lilac alcohol D | 1.8 | ± | 0.4 | 2.0 | ± | 0.6 | 2.6 | ± | 0.5 | 2.1 | ± | 0.7 | 0.8 | ± | 0.3 | 2.5 | ± | 0.7 | 1.7 | ± | 0.4 | 1.0 | ± | 0.4 |
|  | Lilac aldehyde degradation | 0.5 | ± | 0.1 | 0.6 | ± | 0.2 | 0.5 | ± | 0.1 | 0.8 | ± | 0.2 | 0.5 | ± | 0.1 | 0.6 | ± | 0.1 | 0.6 | ± | 0.1 | 0.5 | ± | 0.1 |
| Nitrogen bearing | **2-Methylbutylaldoxime** | 0.9 | ± | 0.9 | 0.0 | ± | 0.0 | 0.0 | ± | 0.0 | 0.0 | ± | 0.0 | 5.2 | ± | 2.8 | 0.8 | ± | 0.8 | 6.3 | ± | 3.3 | 0.0 | ± | 0.0 |
|  | **3-Methylbutanal oxime** | 0.0 | ± | 0.0 | 9.2 | ± | 9.2 | 9.0 | ± | 9.0 | 10.4 | ± | 10.4 | 28.0 | ± | 15.6 | 27.5 | ± | 15.5 | 33.6 | ± | 15.9 | 20.3 | ± | 14.1 |
| Phenol | Cinnamene | 13.2 | ± | 2.6 | 11.9 | ± | 2.2 | 11.4 | ± | 2.0 | 15.9 | ± | 2.9 | 12.1 | ± | 1.9 | 14.5 | ± | 2.1 | 14.2 | ± | 2.3 | 14.3 | ± | 2.3 |
|  | 1,2,3-Trimethylbenzene | 2.7 | ± | 0.5 | 2.6 | ± | 0.7 | 2.4 | ± | 0.6 | 3.6 | ± | 0.7 | 2.1 | ± | 0.5 | 3.0 | ± | 0.7 | 2.8 | ± | 0.6 | 3.0 | ± | 0.6 |
|  | Acetophenone | 9.5 | ± | 1.2 | 10.0 | ± | 1.1 | 9.3 | ± | 0.9 | 9.7 | ± | 1.1 | 9.0 | ± | 1.1 | 8.0 | ± | 1.0 | 8.5 | ± | 1.0 | 11.0 | ± | 1.0 |
| Phenyl-propanoide | **Guaicol** | 1.3 | ± | 0.4 | 1.7 | ± | 1.1 | 3.5 | ± | 1.2 | 2.7 | ± | 0.7 | 11.8 | ± | 6.7 | 8.8 | ± | 3.4 | 11.3 | ± | 4.3 | 3.1 | ± | 1.1 |
|  | **2-Phenylethanol** | 12.9 | ± | 5.2 | 17.3 | ± | 7.2 | 8.7 | ± | 2.3 | 16.0 | ± | 4.4 | 16.9 | ± | 5.5 | 21.2 | ± | 6.6 | 17.4 | ± | 5.0 | 15.5 | ± | 5.9 |
|  | Hydrocinnamyl alcohol | 15.9 | ± | 6.3 | 31.3 | ± | 12.0 | 12.5 | ± | 7.2 | 13.0 | ± | 6.9 | 28.7 | ± | 9.9 | 28.4 | ± | 10.8 | 33.9 | ± | 12.5 | 30.2 | ± | 11.1 |
|  | (E)-Cinnamaldehyde | 4.6 | ± | 4.6 | 5.4 | ± | 5.4 | 0.0 | ± | 0.0 | 0.0 | ± | 0.0 | 0.0 | ± | 0.0 | 0.0 | ± | 0.0 | 2.9 | ± | 2.9 | 0.0 | ± | 0.0 |
|  | (E)-Cinnamyl alcohol | 61.0 | ± | 21.6 | 61.2 | ± | 19.0 | 45.7 | ± | 17.1 | 46.7 | ± | 16.4 | 91.6 | ± | 19.0 | 84.4 | ± | 17.9 | 71.7 | ± | 18.3 | 74.0 | ± | 16.5 |
|  | Methyleugenol | 0.5 | ± | 0.2 | 0.7 | ± | 0.4 | 0.0 | ± | 0.0 | 0.5 | ± | 0.4 | 0.6 | ± | 0.3 | 0.5 | ± | 0.3 | 0.3 | ± | 0.2 | 0.7 | ± | 0.5 |
|  | Benzenepropyl acetate | 0.0 | ± | 0.0 | 2.1 | ± | 1.7 | 0.3 | ± | 0.3 | 0.1 | ± | 0.1 | 0.6 | ± | 0.3 | 1.0 | ± | 0.9 | 2.2 | ± | 1.3 | 0.9 | ± | 0.9 |
|  | (*Z*)-Cinnamyl alcohol acetate | 1.2 | ± | 1.2 | 2.0 | ± | 0.8 | 2.4 | ± | 2.0 | 0.6 | ± | 0.5 | 1.9 | ± | 0.9 | 1.0 | ± | 0.6 | 1.1 | ± | 0.5 | 1.4 | ± | 0.6 |
|  | Benzyl benzoate | 32.7 | ± | 9.7 | 22.2 | ± | 7.8 | 25.0 | ± | 7.8 | 42.0 | ± | 11.0 | 14.7 | ± | 4.2 | 15.7 | ± | 5.5 | 34.7 | ± | 9.8 | 37.8 | ± | 7.1 |
| Sesquiterpene | α-Farnesene | 0.5 | ± | 0.3 | 0.2 | ± | 0.1 | 0.6 | ± | 0.4 | 0.5 | ± | 0.3 | 2.3 | ± | 1.2 | 0.4 | ± | 0.2 | 1.8 | ± | 0.9 | 2.1 | ± | 1.4 |
| Unknown | Unknown_1 | 48.8 | ± | 12.6 | 60.6 | ± | 15.1 | 52.6 | ± | 12.7 | 54.6 | ± | 13.3 | 85.8 | ± | 16.4 | 90.9 | ± | 17.4 | 66.0 | ± | 14.7 | 81.0 | ± | 15.0 |
|  | Unknown_2 | 0.5 | ± | 0.3 | 0.4 | ± | 0.1 | 1.8 | ± | 0.9 | 2.3 | ± | 0.8 | 0.4 | ± | 0.1 | 0.6 | ± | 0.5 | 1.3 | ± | 0.3 | 1.6 | ± | 0.5 |
|  | Unknown_3 | 1.1 | ± | 0.3 | 1.0 | ± | 0.3 | 0.3 | ± | 0.2 | 0.5 | ± | 0.3 | 0.6 | ± | 0.2 | 0.7 | ± | 0.3 | 0.7 | ± | 0.3 | 1.0 | ± | 0.3 |
|  | Unknown_4 | 11.8 | ± | 1.0 | 14.0 | ± | 2.6 | 15.7 | ± | 2.6 | 14.0 | ± | 1.9 | 11.5 | ± | 1.7 | 8.8 | ± | 1.0 | 10.3 | ± | 1.2 | 9.1 | ± | 0.8 |
